# Pacing Characteristics of His Bundle Pacing vs. Left Bundle Branch Pacing: A Systematic Review and Meta-Analysis

**DOI:** 10.3389/fcvm.2022.849143

**Published:** 2022-03-22

**Authors:** Wen Zhuo, Xiaojie Zhong, Hualong Liu, Jianhua Yu, Qi Chen, Jinzhu Hu, Qinmei Xiong, Kui Hong

**Affiliations:** ^1^Department of Cardiovascular Medicine, The Second Affiliated Hospital of Nanchang University, Nanchang, China; ^2^Jiangxi Key Laboratory of Molecular Medicine, Nanchang University, Nanchang, China; ^3^Department of Genetic Medicine, The Second Affiliated Hospital of Nanchang University, Nanchang, China

**Keywords:** his bundle pacing, left bundle branch pacing, prognosis, physiologic pacing, pacing parameters

## Abstract

**Background:**

His bundle pacing (HBP) is a physiological pacing strategy, which aims to capture the His bundle-Purkinje system and synchronously activate the ventricles. Left bundle branch pacing (LBBP) is a newly discovered physiological pacing technique similar to HBP. We conducted this meta-analysis to compare the pacing parameters and clinical results between HBP and LBBP.

**Methods:**

We systematically retrieved studies using the PubMed, Embase database, and Cochrane Library. Mean difference (MD) and relative risk (RR) with their 95% confidence intervals [CIs] were used to measure the outcomes. A random-effect model was used when studies were of high heterogeneity.

**Results:**

A total of seven studies containing 867 individuals were included. Compared with HBP, LBBP was associated with higher implant success rates (RR: 1.12, 95% CI: 1.05–1.18; *I*^2^ = 60%, *P* = 0.0003), lower capture threshold at implantation (V/0.5 ms) (MD: 0.63, 95% CI: 0.35–0.90, *I*^2^ = 89%, *P* < 0.0001) and capture threshold at follow-up (V/0.5 ms) (MD: 0.76, 95% CI: 0.34–1.18, *I*^2^ = 93%, *P* = 0.0004), and larger sensed R wave amplitude (mV) at implantation (MD: 7.23, 95% CI: 5.29–9.16, *P* < 0.0001) and sensed R wave amplitude (mV) at follow-up (MD: 7.53, 95% CI: 6.85–8.22, *P* < 0.0001). In LBBP recipients, greater QRS wave complex reduction was found in the paced QRS duration at follow-up compared with HBP recipients at follow-up (MD: 6.12, 95% CI: 1.23–11.01, *I*^2^ = 0%, *P* = 0.01). No statistical differences were found in procedure duration, fluoroscopy time, native left ventricular ejection fractions (LVEF), LVEF improvement, native QRS duration, and QRS reduction from the native QRS duration vs. paced QRS duration at implantation.

**Conclusion:**

Current evidence suggests that pacing characteristics are better in LBBP compared with HBP. Further prospective studies are needed to validate the clinical advantages of LBBP.

## Introduction

Traditional right ventricular pacing (RVP), including right ventricular apical, septal, or outflow tract pacing, does not result in synchronous ventricular activation and contraction. It is associated with an increased risk of ventricular remodeling and atrial fibrillation (AF) and can lead to left ventricular dysfunction (1–4).

The development of biventricular pacing (BVP) may have a beneficial hemodynamic effect on patients with left bundle branch block and can improve the prognosis of patients with symptomatic heart failure (5, 6). Despite BVP significantly improving prognosis compared with RVP (7), 1/3 of patients with BVP indications do not gain significant clinical benefit after treatment (8, 9).

His bundle pacing (HBP), by capturing and conducting *via* His bundle-Purkinje fibers and then by activating the ventricle from the normal physiological sequence, is considered to be the most physiological pacing strategy (10). It is effective in the treatment of bradycardia arrhythmias and chronic AF with heart failure. However, there are still some limitations with HBP, such as long fluoroscopy times, high pacing thresholds, and high incidences of early battery depletion and lead dislodgement (11). Recently, Huang et al. reported left bundle branch pacing (LBBP) as an alternative to HBP (12). LBBP has a physiological pacing effect similar to HBP, and some studies have evaluated its safety and effectiveness (13–15).

Currently, few clinical studies are comparing HBP with LBBP. The purpose of this meta-analysis is to further analyze the current clinical studies, comparing pacing parameters, clinical safety, and efficacy of HBP vs. LBBP.

## Methods

The study was prepared according to the Preferred Reporting Items for Systematic Reviews and Meta-Analyses (PRISMA) guidelines (16).

### Search Strategy

We systematically searched relevant studies in PubMed, Embase database, and Cochrane Library up until October 15, 2021. No language or publication restrictions were placed. The MeSH terms and free text words were used to retrieve studies. The first group of keywords was linked to HBP (“His bundle pacing” or “Hisian pacing” or “para-His bundle pacing” or “para-Hisian pacing”). The second group of keywords was linked to LBBP (“left bundle branch pacing” or “left bundle branch area pacing”). The two groups of keywords were combined using the Boolean operator “AND.” The studies were selected independently by two reviewers (Wen Zhuo and Xiaojie Zhong). Endnote X8 was used to manage the studies. These two authors independently reviewed the title and abstract and excluded the irrelevant studies. Full texts were further scrutinized to assess whether the studies could be included in the meta-analysis. The controversy was resolved by discussion or consultation with additional coauthors (Qinmei Xiong and Jinzhu Hu).

### Selection Criteria

Eligible studies that focused on a direct comparison between HBP and LBBP were included in line with the following criteria: (1) published in English with an available full text; (2) measurable parameters have been reported, such as implantation success rates, procedure duration, fluoroscopy time, QRS duration, left ventricular ejection fractions (LVEF), sensed R wave amplitude, or capture threshold; and (3) the follow-up duration was at least 3 months.

Studies were excluded if (1) they were certain publication types, such as reviews, meta-analyses, notes, case reports, or conference abstracts; (2) they had overlapping study populations; or (3) the full text was unavailable.

### Data Extraction and Quality Assessment

The data were extracted independently by two authors (Wen Zhuo and Hualong Liu) on a standard data extraction form. The following information was extracted from the studies: author's name, publication year, study design, country, sample size, follow-up duration, age, sex ratio, number of participants, primary diseases, procedure duration, fluoroscopy time, native LVEF, LVEF at follow-up, native QRS duration, QRS duration at implantation and follow-up, sensed R wave amplitude at implantation and follow-up, and capture threshold at implantation and follow-up. The quality of included studies was assessed using the Newcastle-Ottawa Scale (NOS). Each study was scored based on selection, comparability, and outcome by two reviewers independently. One star would be given to a positive response to one question on the scale. The maximum number of stars each article could get was 9. We considered a study with a NOS score >6 stars to be of high quality.

### Outcomes

The procedural outcomes included the implant success rates, procedure duration, and fluoroscopy time. The efficacy outcomes included QRS duration reduction (native QRS duration minus paced QRS duration at implantation and native QRS duration minus paced QRS duration at follow-up), sensed R wave amplitude at implantation, sensed R wave amplitude at follow-up, paced capture threshold at implantation, paced capture threshold at follow-up, native LVEF, and LVEF improvement (LVEF at follow-up minus native LVEF).

### Statistical Analysis

We used Review Manager 5.3 (Cochrane Collaboration, Copenhagen, Denmark) to perform our meta-analysis. Mean difference (MD) was used for the outcomes of continuous variables, whereas relative risk (RR) was used for the categorized variables. The 95% confidence intervals (CIs) for MD and RR were also calculated. Heterogeneity among studies was assessed using chi-squared and I-squared tests. A *P* < 0.10 was considered to indicate the existence of heterogeneity among the studies. We considered substantial heterogeneity to exist when *I*^2^ > 50% and a random-effect model was used. Otherwise, a fixed-effect model was used.

## Results

### Literature Search

**Figure 1:**
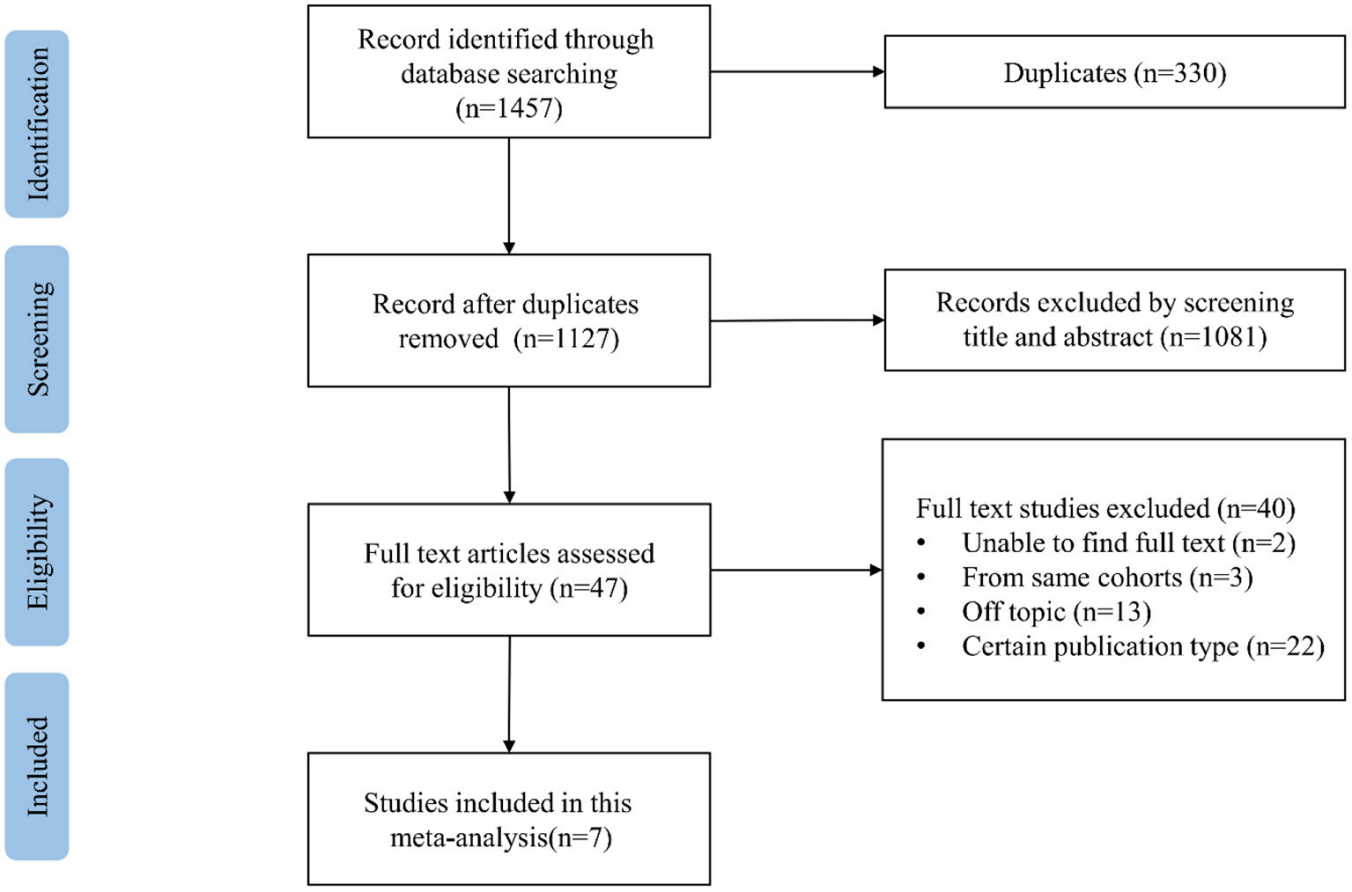
Flowchart of study selection.

Our search results are summarized in the PRISMA flowchart (Supplementary Table). The process of literature searching is shown in Figure 1. We initially identified 1,457 potential data sources through electronic retrieval strategies with 330 duplicates. After reviewing the titles and abstracts, 47 studies were qualified for a full review. Then intensive reading was done, and 40 studies were excluded, among which 13 articles were off-topic, two studies were without full text, and 3 studies had overlapping study populations. Among the studies describing the same cohort, we selected the studies including the largest number of participants. A total of 22 articles were excluded by publication type, including eight case-report studies, four review studies, and 10 conference abstracts. Finally, seven studies were found to be eligible for the meta-analysis (15, 17–22).

### Study Characteristics

A total number of 867 individuals were included for analysis. Among them, 476 were men, and the estimated mean age of all individuals was 68.8 ± 12.9 years. Table 1 provides the main characteristics and the relevant parameters of the included studies. Basic parameters of clinical studies were extracted, such as author's name, year of publication, regions, study design, age, sex, follow-up duration, number of participants, and primary diseases. The quality of the included studies was high, with NOS scores varying from 8 to 9, and the results are shown in Table 2.

**Table 1:** Basic characteristics of studies included in the meta-analysis.

**References**	**Study design**	**Region**	**Number of participants (*N*)**	**Follow-Up duration**	**Age (year)**	**Male (%)**	**Disease**	**Date of included patients**	**Implant success (%)**
Hou et al. (15)	Prospective study	China	HBP: 29; LBBP:56	1/6 M	HBP:69.1 ± 10.4; LBBP: 68.3 ± 11.8	HBP:65.5; LBBP: 64.3	SND/AVB/AF	2018.1–2018.9	Not mentioned
Hua et al. (17)	Retrospective study	China	HBP:125; LBBP:126	3 M	HBP:62.2 ± 15.2; LBBP: 65.3 ± 11.1	HBP:56.8; LBBP:46	Bradycardia	2018.1–2019.4	HBP:87.2%; LBBP:91.3%
Molina-Lerma et al. (18)	Retrospective study	Spain	HBP:45; LBBP:42	3 M	HBP:75.5; LBBP:76	HBP:62.2; LBBP:59.5	Not mentioned	HBP:2018.1–2018.12; LBBP:2019.1–2019.12	Not mentioned
Qian et al. (19)	Retrospective study	China	HBP:64; LBBP:185	3/6 M/1 Y	HBP:66.7 ± 10.8; LBBP:68.9 ± 12.5	HBP:59.4; LBBP:55.1	Bradycardia/HF	2014.9–2019.8	HBP:87.6%; LBBP:95.9%
Sheng et al. (20)	Retrospective study	China	26	3 M	72.9 ± 9.0	65.4	Bradycardia/AF	2019.1–2019.6	Not mentioned
Vijayaraman (21)	Retrospective study	Multiple centers	HBP:46; LBBP:28	12.0 ± 13.7 M	79 ± 8	57	Not mentioned	Not mentioned	HBP:63%; LBBP:93%
Wu (22)	Prospective, non-randomized study	China	HBP:49; LBBP:32	1 Y	HBP:68.3 ± 10; LBBP:67.2 ± 13	HBP:63.3; LBBP:43.8	LBBB/HF/CRT recipients	2012.12–2018.12	HBP:99.2%; LBBP:98.9%

*HBP, his-bundle pacing; LBBP, left bundle branch pacing; AF, atrial fibrillation; AVB, atrioventricular block; SND, sinus node dysfunction; HF, heart failure; LBBB, left bundle branch blocked*.

**Table 2:** Quality assessment based on the Newcastle–Ottawa scale.

**Study**	**Representativeness of the patient**	**Selection of the controls**	**Ascertainment Of intervention**	**Demonstration that outcome of interest was not present at the start of the study**	**Comparability-age and gender**	**Comparability-Other factors**	**Assessment of outcome**	**Was follow-up long enough for outcomes to occur**	**Adequacy of follow-up of cohorts**	**Total**
1. Hua et al. (17)	1	1	1	1	1	1	1	0	1	8
2. Molina-Lerma et al. (18)	1	1	1	1	1	1	1	0	1	8
3. Qian et al. (19)	1	1	1	1	1	1	1	1	1	9
4. Sheng et al. (20)	1	1	1	1	1	1	1	0	1	8
5. Vijayaraman (21)	1	1	1	1	1	1	1	1	1	9
6. Wu (22)	1	1	1	1	1	1	1	1	1	9
7. Hou et al. (15)	1	1	1	1	1	1	1	1	1	9

*Average score: 8.57*.

### Procedure Assessment

**Figure 2:**
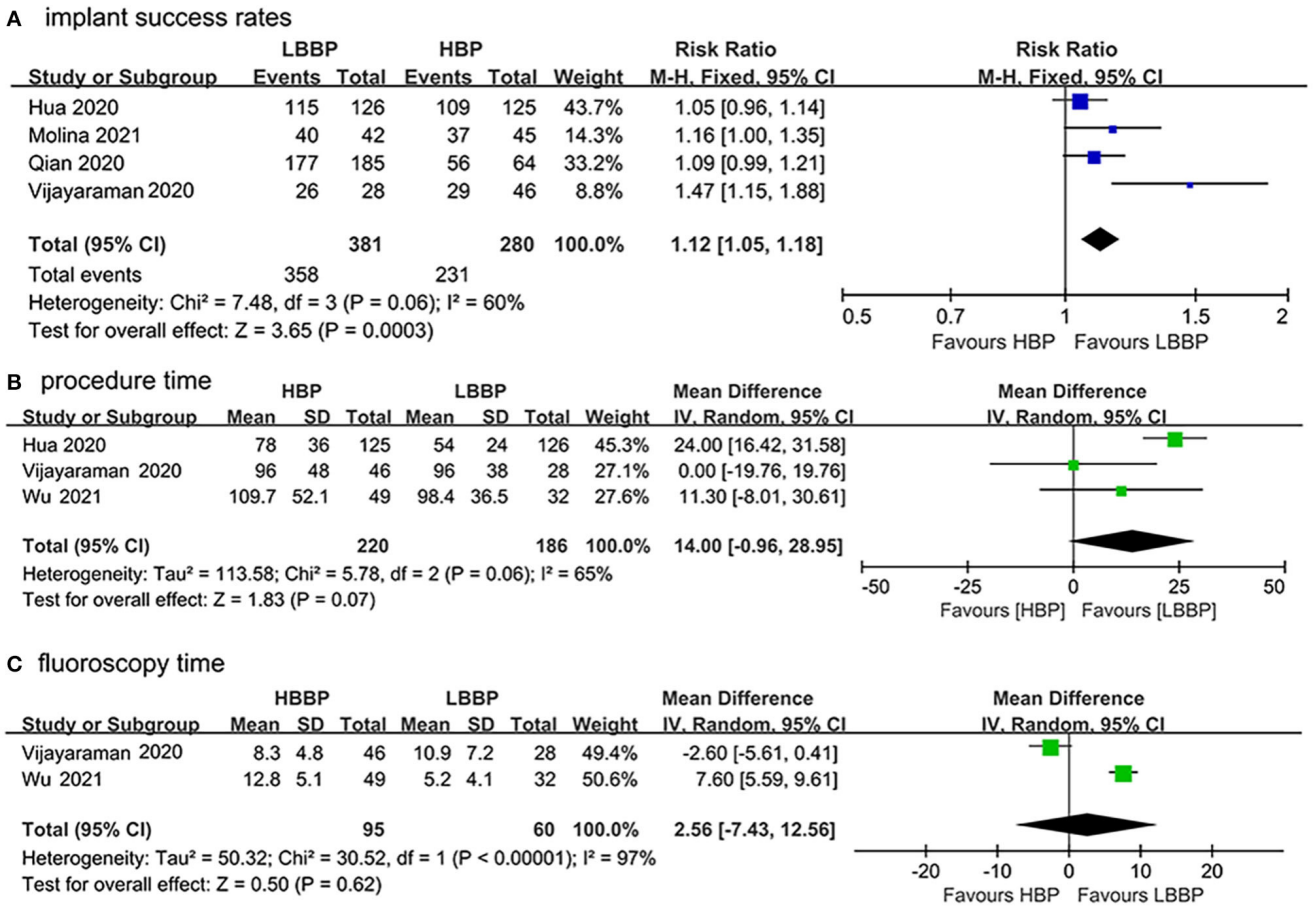
Procedural outcomes of HBP vs. LBBP **(A)** implant success rates, **(B)** procedure duration, and **(C)** fluoroscopy time.

Data from the four included studies (17–19, 21) were extracted to analyze the implant success rates. As shown in Figure 2A, LBBP was associated with a statistically significant higher success rate compared with HBP (RR: 1.12, 95%CI: 1.05–1.18; *I*^2^ = 60%, *P* = 0.0003). In total, three included studies (17, 21, 22) measured the mean procedure duration, and two studies (21, 22) reported the fluoroscopy time. No statistical difference was observed in the procedure duration between patients who received HBP or LBBP (MD: 14.00, 95% CI: −0.96–28.95, *I*^2^ = 65%, *P* = 0.07; Figure 2B). There was no significant difference in fluoroscopy time between HBP or LBBP recipients (MD: 2.56, 95% CI: −7.43–12.56, *I*^2^ = 97%, *P* = 0.62; Figure 2C). Due to the existence of heterogeneity, a random model was used.

In total, four studies (18, 19, 21, 22) reported surgical complications on at least one of the following: lead dislodgement, loss of capture, macro displacement, increase in pacing threshold, and pocket infection. Kaplan–Meier estimates for overall complication rate were not analyzed due to lack of data.

### Efficacy Assessment

#### R-Wave Amplitude

**Figure 3:**
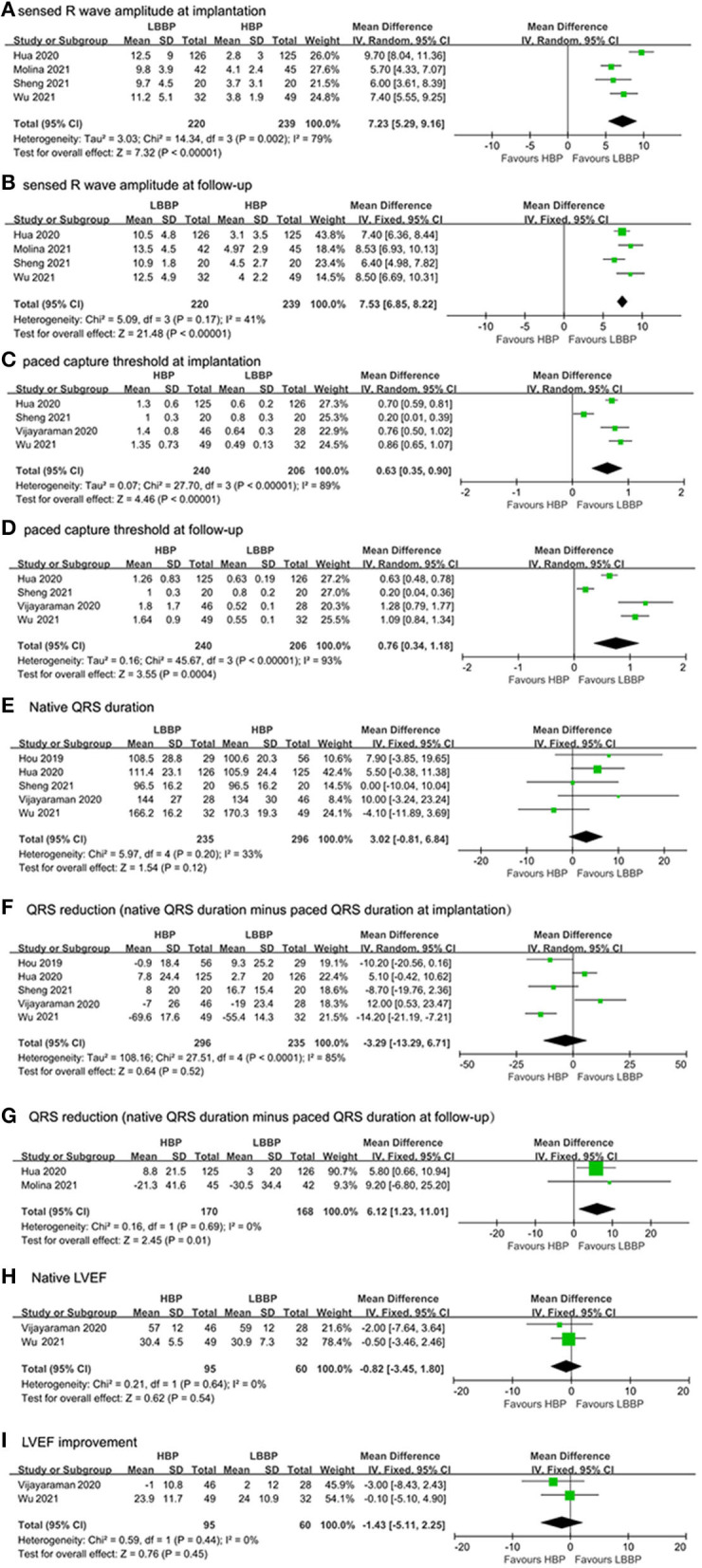
Efficacy characteristics of implantation outcomes and surgical complications: **(A)** capture threshold at implantation, **(B)** capture threshold at follow-up, **(C)** sensed R wave amplitude at implantation, **(D)** sensed R wave amplitude at follow-up, **(E)** native QRS duration, **(F)** QRS duration reduction (native QRS duration minus paced QRS duration at implantation), **(G)** QRS duration reduction (native QRS duration minus paced QRS duration at follow-up), **(H)** native LVEF, and **(I)** LVEF improvement.

In total, four studies (17, 18, 20, 22) reported R wave amplitudes. As shown in Figure 3A, our study found that the sensed R wave amplitude at implantation of LBBP recipients was larger than HBP recipients (MD: 7.23, 95% CI: 5.29–9.16, *P* < 0.0001). Due to the existence of significant heterogeneity (*I*^2^ = 79%), a random model was used. The sensed R wave amplitude at follow-up was also assessed in HBP and LBBP recipients; the results also showed that R wave amplitude was larger in LBBP recipients than HBP recipients (MD: 7.53, 95% CI: 6.85–8.22, *P* < 0.0001; Figure 3B). The heterogeneity among these studies was low (*I*^2^ = 41%), and a fixed model was used.

#### Capture Threshold

In total, four studies (17, 20–22) reported the paced capture threshold at implantation and follow-up. A statistically significant difference was observed at implantation and follow-up in capture threshold. Pooled results showed that capture threshold was lower in patients with LBBP at implantation (MD: 0.63, 95% CI: 0.35–0.90, *I*^2^ = 89%; *P* < 0.0001, Figure 3C) and follow-up (MD: 0.76, 95% CI: 0.34–1.18, *I*^2^ = 93%, *P* = 0.0004; Figure 3D).

#### Reduction of QRS Duration

QRS duration was evaluated in six studies, including five studies (15, 17, 20–22) that reported the native QRS duration and the paced QRS duration at implantation and two studies (17, 18) that reported the native QRS duration and the paced QRS duration at follow-up.

We compared QRS duration reduction in our meta-analysis by subtracting paced QRS duration at implantation from the native QRS duration and subtracting paced QRS duration from the native QRS duration at follow-up. As shown in Figures 3E,F, no statistical difference was observed in native QRS duration and the reduction of QRS duration between paced QRS duration at implantation (MD: 3.02, 95% CI: −0.81–6.84, *I*^2^ = 33%, *P* = 0.12) and native QRS duration (MD: −3.29, 95% CI: −13.29–6.71, *I*^2^ = 85%, *P* = 0.52). In LBBP recipients, greater QRS reduction was found in the paced QRS duration at follow-up compared with HBP recipients (MD: 6.12, 95% CI: 1.23–11.01, *I*^2^ = 0%, *P* = 0.01; Figure 3G).

#### Left Ventricular Ejection Fractions

In total, two studies (21, 22) reported the LVEF values at baseline and after follow-up to assess the cardiac function of HBP and LBBP recipients. Figure 3H shows no statistical difference in native LVEF between HBP and LBBP recipients (MD: −0.82, 95% CI: −3.45–1.80, *P* = 0.45). As shown in Figure 3I, no statistical difference in LVEF improvement was found between HBP and LBBP recipients (MD: −1.43, 95% CI: −5.11–2.25, *P* = 0.45). There was no heterogeneity among these studies (*I*^2^ = 0%).

## Discussion

In this meta-analysis, it can be observed that LBBP is associated with a higher implant success rate than HBP, and the QRS duration was shorter after follow-up compared with native QRS duration. Second, data show that LBBP recipients have larger R wave amplitudes and lower capture thresholds than HBP recipients postoperatively and after follow-up, while no statistically significant difference in reduction of QRS duration was found between these pacing modalities at baseline. Other pacing parameters and clinical characteristics did not differ significantly between LBBP and HBP.

Since no randomized controlled trials (RCTs) have been published comparing the assessment of the safety and effectiveness of HBP and LBBP, our findings provide some evidence that compared with HBP, LBBP may be easier to implant and has better pacing parameters including capture threshold and R wave amplitude.

Although HBP provides physiological pacing and benefits many patients, it still has some limitations in practice. In addition to electrogram recordings, defining the anatomical region of the HB requires fluoroscopy. When patients have anatomical variations or enlarged right atria, locating the area of the HB can be challenging, which complicates implantation. In some instances, it is difficult to determine where to screw in the lead as well as whether the depth and direction of the lead are appropriate. Optimal lead placement may depend on the use of three-dimensional electroanatomic mapping and/or intracavitary ultrasound (23). The LBB is widely distributed below the left septal endocardium, making it easier to place the lead and capture the left ventricular conduction system (24). These aspects might explain the higher success rate of LBBP.

Previous studies have shown that the HBP capture threshold is significantly higher than the traditional RVP capture threshold (11, 25), which will lead to faster battery depletion and more frequent lead revisions. The exact mechanism is not clear, which may be related to the lead relaxation caused by tricuspid valve movement, inadequate lead fixation, and local fibrosis of the tissue around the lead. However, LBBP can maintain a low pacing threshold during the follow-up period and show higher R wave amplitude (11, 14, 26). This can be explained by noting that the LBBP lead is positioned deep within the left ventricular septum and close to the myocardial tissue, stimulating not only the specialized conduction system but also the deep myocardium of the interventricular septum. Of note, Kawashima et al. found three variations in His bundle anatomy (27), showing that 79% of His bundle are insulated by myocardial fibers, suggesting that the low amplitude of R wave in HBP may be related to the myocardial limitations around the HB region. His bundle encapsulation by myocardial fibrous sheaths may be linked to high capture thresholds during HBP. Our findings show that LBBP has a higher R wave amplitude and a lower capture threshold than HBP, which is consistent with the above studies.

QRS duration is an important indicator of ventricular systolic synchronization in ECG parameters. HBP keeps the electromechanical activity of left and right ventricles synchronized, showing a narrow QRS duration on ECG. The typical pacing QRS morphology of LBBP is characterized by an incomplete right bundle branch block pattern, resulting in longer pacing QRS duration than intrinsic QRS duration (28). However, LBBP may also lead to a narrow QRS duration due to the activation of the right bundle branch by retrograde conduction, intrinsic conduction fusion, and the communications between the bundle branches (29).

In this study, we first analyzed the QRS duration reduction by subtracting paced QRS at implantation from the native QRS duration. In total, three studies reported prolonged QRS duration by LBBP (15, 17, 20), and two studies reported shortened QRS duration by LBBP (21, 22). The final combined results show that there is no statistical difference in the changes in QRS duration between HBP and LBBP at implantation. Then we analyzed QRS duration reduction by subtracting paced QRS duration at follow-up from the native QRS duration, and our results demonstrated that QRS duration reduction from LBBP is greater than that of HBP at follow-up. It can also be interpreted that LBBP recipients have a lower rate of lead dislodgement, suggesting that the long-term stability of LBBP is better than that of HBP. However, the limited data make it hard to confirm the better performance of LBBP than HBP, and more studies are needed for further verification.

Several studies have shown that HBP and LBBP can improve the LVEF of patients (12, 14, 30, 31). Our results showed no statistical difference in the improvement of LVEF between HBP and LBBP, and both pacing modes had a positive effect on patients with left ventricular dysfunction, indicating that despite LBBP demonstrating better pacing parameters, HBP is not inferior to LBBP in improving cardiac function.

Other pacing parameters, including the mean procedure time and fluoroscopy time, were not statistically different between LBBP and HBP due to the small number of included studies. As for the fluoroscopy time, Vijayaraman et al. (21) had a longer fluoroscopy time in LBBP than in HBP, contrary to Wu et al. (22). The learning curve of HBP has shown that procedure time and fluoroscopy time were shorter with increasing operator experience (32). The differences in procedure time and fluoroscopy time in different studies may be related to the skills and experience of different operators.

## Limitations

Our meta-analysis has several limitations, and the results should be interpreted with caution. First, the included studies are observational cohort studies with small sample sizes rather than randomized controlled trials. Second, owing to only two studies reporting the QRS duration at baseline and follow-up, the real relationship of QRS duration reduction at baseline and follow-up among HBP or LBBP recipients needs to be further investigated. Third, QRS morphology could not be analyzed because of limited reporting in the included studies. Fourth, the primary disease of LBBP or HBP recipients could not be distinguished due to a lack of data. Fifth, as LBBP is a newly discovered pacing technique, the included studies had a short follow-up duration, no longer than 1 year, and long-term outcomes are unavailable. Consequently, multicenter, double-blinded RCTs are still needed to validate the clinical advantages of LBBP.

## Conclusion

This meta-analysis has shown that compared with HBP, LBBP is associated with a higher implant success rate, larger R wave amplitude, and lower capture threshold. Pacing characteristics are better with LBBP compared with HBP. LBBP appears to be a promising, possibly superior, and alternative to HBP.

## Data Availability Statement

The original contributions presented in the study are included in the article/Supplementary Material, further inquiries can be directed to the corresponding authors.

## Author Contributions

HL, JY, QC, JH, QX, and KH: supervision. QX and KH: conceptualisation and formal analysis. HL, JY, QC, JH, QX, XZ, and KH: validation, writing—review, and editing. WZ and XZ: investigation. XZ: data curation. WZ: formal analysis, writing—original draft, visualization, and project administration. All authors contributed to the article and approved the submitted version.

## Funding

This study was funded by the Science and Technology Research Project of the Education Department of Jiangxi Province (200192) and Science and Technology Plan of Jiangxi Provincial Health Commission (202130420).

## Conflict of Interest

The authors declare that the research was conducted in the absence of any commercial or financial relationships that could be construed as a potential conflict of interest.

## Publisher's Note

All claims expressed in this article are solely those of the authors and do not necessarily represent those of their affiliated organizations, or those of the publisher, the editors and the reviewers. Any product that may be evaluated in this article, or claim that may be made by its manufacturer, is not guaranteed or endorsed by the publisher.

## Abbreviations

HBP: His bundle pacing
LBBP: Left bundle branch pacing
MD: Mean difference
CIs: Confidence intervals
RR: Relative risk
AF: Atrial fibrillation
RVP: Right ventricular pacing
BVP: Biventricular pacing
PRISMA: Preferred Reporting Items for Systematic Reviews and Meta-Analyses
LVEF: Left ventricular ejection fractions
NOS: Newcastle-Ottawa Scale
MeSH: Medical Subject Headings
RevMan: Review Manager.
